# Natural Products as a Novel Therapeutic Strategy for NLRP3 Inflammasome-Mediated Gout

**DOI:** 10.3389/fphar.2022.861399

**Published:** 2022-03-16

**Authors:** Jun Ho Lee, Hyuk Soon Kim, Jun-Ho Lee, Gabsik Yang, Hong Jun Kim

**Affiliations:** ^1^ College of Korea Medicine, Woosuk University, Jeonju-si, South Korea; ^2^ Department of Biomedical Sciences, College of Natural Science and Department of Health Sciences, The Graduate School of Dong-A University, Busan, South Korea

**Keywords:** gout, monosodium urate (MSU), NLRP3 inflammasome, interleukin-1β, natural producct

## Abstract

Gout is the most common form of inflammatory arthritis. It occurs when monosodium urate crystals (MSU) are deposited within joints due to hyperuricemia and persistent elevations of serum uric acid levels. Traditional gout treatment such as urate-lowering therapy is difficult to continue for a long period of time due to the risk of side effects. Recent studies have shown that the modulation of MSU-induced inflammatory responses is dependent on the inflammatory cytokine IL-1β, which has a central role in a chain of processes involving multiple cytokines and mediators. In this regard, the NLRP3 inflammasome is known to play a crucial part and thus has been proposed as a novel target in the treatment for gout. However, the biochemical mechanism for NLRP3 inflammasome activation has not yet been clearly elucidated. Therefore, this report can provide an overview of natural extractions targeted to prevent or treat NLRP3 inflammasome-mediated gout in the MSU-induced gout model. In addition, the research and development of such natural products are suggested as a potential strategy in the treatment of gout.

## Introduction

Gout is the most common form of inflammatory arthritis affecting adults. It is a chronic disease caused by monosodium urate (MSU) crystal deposition. A recent study reported the range from prevalence as <1%–6.8% and the incidence as 0.58–2.89 per 1,000 person-years (Dehlin et al., 2020). Gout typically presents as an acute, self-limited inflammatory monoarthritis affecting the joints of the lower extremities. Elevated serum uric acid level (hyperuricemia) is a major risk factor for MSU crystal deposition and the onset of gout. MSU crystals are preferentially deposited in the joints and periarticular structures. In particular, gout usually presents as acute, excruciating pain, and inflammatory arthritis in the first metatarsophalangeal joint, metatarsophagus, and knee area (Taylor et al., 2015). Gout flares usually resolve naturally within 7–10 days and are interspersed between asymptomatic ‘intercritical phases.’ Over time, prolonged hyperuricemia can cause more frequent and severe redness that also affects the upper extremities and several joints (Gutman, 1973). In Traditional Korean Medicine, terminology such as bibing, lijiefeng, baihufeng, and tongfeng are used to describe disorders with comparable characteristics to gout. Among these, the ‘tongfeng’ notion dates all the way back to the Jin and Yuan eras. Throughout the Ming and Qing dynasties, different theories on the etiology, prevention, and treatment of tongfeng were suggested (Cho and Jung, 2015). Acupuncture with bee venom has anti-inflammatory and analgesic properties, and it has long been utilized as a traditional Korean therapy for musculoskeletal problems, particularly gout arthritis (Goo et al., 2021).

Currently, in gout patients, pharmacological agents that reduce purine break-down (i.e., xanthine oxidase inhibitors (XOIs)) or drugs that increase urinary excretion of uric acid (i.e., uric acid preparations) are generally administered as urate-lowering therapy to decrease uric acid levels. Allopurinol is the oldest XOI currently in use, and more recently Febuxostat, which has a more selective and less complex dosing regimen than allopurinol, is also being administered to patients. Additionally, marketed uric acid excretion agents that increase uric acid excretion primarily through urine include probenecid, sulfinpyrazone and benzbromarone, as well as the recently approved lesinurad (Dalbeth et al., 2017; Saag et al., 2017). However, XOIs have disadvantages such as multiple dose regimens, unsuitability for use in patients with severe chronic kidney disease, and increased risk of renal calculus formation. For this reason, researchers have been exploring alternative treatment strategies such as those involving the use of natural products.

On the other hand, pathologically, joints affected by gout flare are characterized by distinct neutrophil infiltration in both the synovial tissue and synovial fluid (Landis and Haskard, 2001). Recent studies have shown that interleukin-1β (IL-1β) is a major regulatory inflammatory cytokine of gout, promoting neutrophil influx into the synovial membrane and joint fluid, which is a pathological hallmark of acute inflammatory attacks (Silvestre et al., 2020). Strong evidence for the role of IL-1β in gout-related pain and inflammation is provided in animal and human studies. In a rat gout model, after injection of MSU into the mouse ankle joint, inflammation was significantly reduced in both IL-1 receptor-deficient mice and wild-type mice treated with the IL-1 inhibitor IL1 trap (rilonacept) (Martin et al., 2009; Torres et al., 2009). Therefore, it supports the argument of the pivotal role of IL-1β in the pathogenesis of gout.

In this review, we present background information on gout and the activation of NLR family pyrin domain containing 3 (NLRP3) inflammasome. We also provide research information on natural extracts with NLRP3 inflammasome regulation that have been identified in animal models of MSU-induced gout within the recent 5 years.

## Mechanism of NLRP3 Inflammasome

**FIGURE 1:**
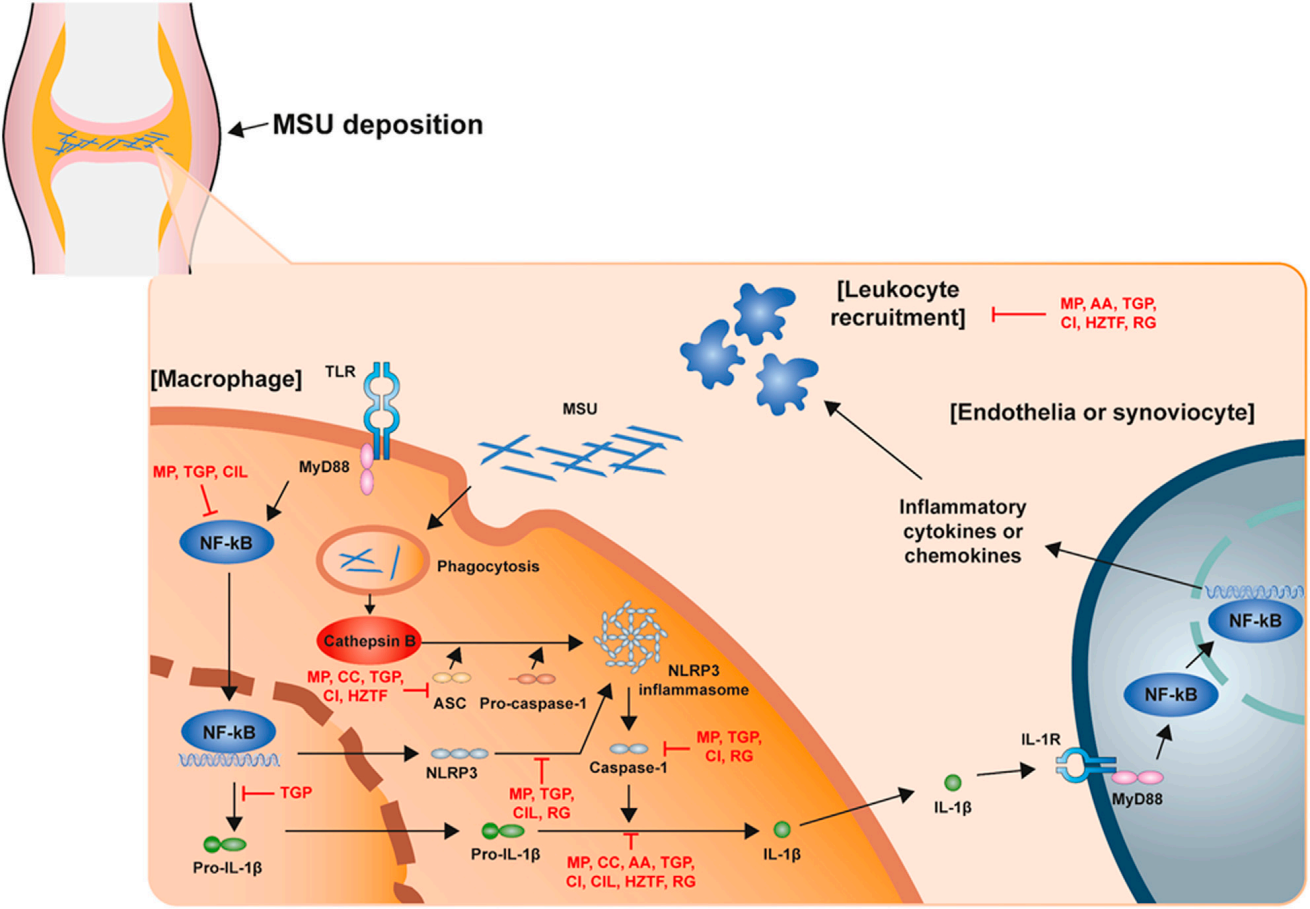
Schematic structure of the acute gout inflammation. Mechanism of NLRP3 inflammasome activation in acute gout inflammation with potential intervention of natural products.

The NLRP3 inflammasome is the most recently studied inflammatory regulatory complex (Lamkanfi and Dixit, 2012; Sutterwala et al., 2014; Elliott and Sutterwala, 2015; He et al., 2016a; Swanson et al., 2019). It is activated by numerous risk-related molecular patterns, including bacterial cell wall components, bacterial RNA or the bacteria themselves, and damage-related molecular patterns, such as free fatty acids, contributing to the pathology of inflammatory diseases. Despite various studies over a long period of time, the mechanism of NLRP3 inflammasome activation is still unclear. However, it is clearly known that NLRP3 inflammasome activation requires two signaling steps provided by several exogenous and endogenous activators. The first signal (priming signal) is nuclear factor kappa B (NF-κB)-dependent transcription of NLRP3 and pro-IL-1 which is triggered by the binding of lipopolysaccharide (LPS) to the Toll-like receptor (TLR) four ligand receptor (Bauernfeind et al., 2009; Muñoz-Planillo et al., 2013; Lamkanfi and Dixit, 2014). The signal then promotes the expression of inflammasome components, including NLRP3, procaspase-1, and pro-IL-1β. The first signal is initiated by TLR4 and then transduced by related adapter molecules, including myeloid differentiation factor 88 (MyD88), IRAK1, and IRAK4, without the requirement for new protein synthesis (Juliana et al., 2012; Fernandes-Alnemri et al., 2013; Lin et al., 2014). The priming signal establish NLRP3 to form inflammasome assembly from degradation by licensing the proteins to form the correct morphology for self-oligomerization and interaction with ASC, through various post-translational modifications including ubiquitylation, deubiquitination, phosphorylation, and sumoylation to NLRP3 (Shim and Lee, 2018; Seok et al., 2020). The second signal (activation signal) involved in the assembly and activation of the NLRP3 complex (the activation signal) is induced by extracellular adenosine triphosphate (ATP), certain bacterial toxins, and various types of crystalline and particulate matter. This signal then produces active cleaved caspa-se-1. When macrophages are activated by NLRP3 activators as part of a second signal, NLRP3 undergoes oligomerization through homologous NACHT domain interactions, which recruit PYD domains to interact with PYDs in ASCs that trigger ASC fibrillar binding. Next, ASC aggregates recruit CARDs and interact with the CARD domain of procaspase-1 to promote caspase-1 activation and subsequent polymerization of ASC fibrils to the ASC spec (Masumoto et al., 1999; Lu et al., 2014). Clustered procaspase-1 self-cleaves and activates in the form of activated caspase-1, which cleaves precursors of IL-1β and IL-18 to produce activated forms of IL-1β and IL-18 (Figure 1), causing an inflammatory response and pyroptosis. GSDMD activated by caspase-1 and -11 forms GSDMD pores in the cell membrane and secretes IL-1beta matured by caspase-1 along with calcium influx (Tsuchiya et al., 2021). In this process, post-translational modifications of NLRP3, such as phosphorylation or deubiquitination, are required for the assembly and activation of the NLRP3 inflammasome (second signal) (Juliana et al., 2012; Lopez-Castejon et al., 2013). In addition, potassium efflux is also a major upstream signaling event for NLRP3 inflammasome activation. Most NLRP3 stimuli is known that can cause the outflow of potassium from cells whereas the inhibition of potassium efflux by high extracellular potassium concentration can block most stimulus-induced NLRP3 inflammasome activation. Recent study demonstrate that Nek7 is an essential protein that acts downstream of potassium efflux to mediate NLRP3 inflammasome oligomerization and activation via structural formation of Nek7-NLRP3 binding (He et al., 2016b; Sharif et al., 2019). However, the exact mechanism by which NLRP3 inflammasome assembly is promoted is still not clearly known. When activation of NLRP3 is triggered, the formation of ASC spec can be considered as an upstream indication of NLRP3 activation (Stutz et al., 2013).

## Role of NLRP3 Inflammasome in Gout

The pathological hallmark of gout attacks is known as neutrophil influx into the synovium and joint fluid (Landis and Haskard, 2001). However, neutrophil does not exist in normal joints, and the interaction between MSU and resident joint cells, which are mainly synovial lining cells, is known to trigger neutrophil influx due to MSU precipitation in the joint. Recent studies show that mononuclear phagocytes play a central role in the initial response to MSU precipitation. When monocyte cells were exposed to MSU, secretion of inflammatory cytokines increased, especially IL-1β, indicating that phagocytosis of MSU crystals was centrally mediated (Yagnik et al., 2000; Landis et al., 2002). Accordingly, Martinon et al. demonstrated that the NLRP3 inflammasome is important for sensing MSU deposition and the subsequent innate immune response (Martinon et al., 2006). Macrophages from mice lacking various components of the inflammasome, including caspase-1, ASC and NLRP3, were unable to activate IL-1β in response to MSU stimulation. Notably, these mice also showed impaired neutrophil influx after intraperitoneal MSU injection, demonstrating that the NLRP3 inflammasome is an important link between the well-established causal stimulation of gout and the subsequent pathological features of acute gout attacks (Martinon et al., 2006; Amaral et al., 2012). In addition to intracellular NLRP3 receptors, TLR2 and TLR4 receptors may also play a role in the innate immune response to MSU deposition (Liu-Bryan et al., 2005). Macrophages isolated from TLR2−/− and TLR4−/− mice showed abnormal uptake of MSU crystals and decreased production of inflammatory cytokines, suggesting that TLR is essential for MSU crystal-induced inflammation.

Although much progress has been made in elucidating the biological pathways that mediate gout attacks, the precise mechanism by which endocytosed MSU crystals activate the NLRP3 inflammasome remains unclear. A recent study of the biological mechanism supporting the association between excessive food intake and the onset of gout demonstrated a synergistic relationship between free fatty acids (FFA) and MSU for IL-1β activation (Joosten et al., 2010). In this study, MSU crystals alone were unable to induce IL-1β release in peripheral blood mononuclear cells (PBMCs) isolated from healthy subjects, whereas large amounts of active IL-1β were detected in the presence of FFA. Notably, IL-1β release following MSU and FFA injection was significantly reduced in caspase-1 and ASC deficient mice, but not in NLRP3 gene deficient mice. In addition, another research suggested that TLR2 and TLR4 are nonessential for the innate immune response to MSU crystals in the peritoneal model of acute gout (Liu-Bryan et al., 2005). However, it can also be considered that the response to MSU stimulation through different mechanisms occurrs in cells of different tissues.

Although pathologically it has been firmly established that the activation of the NLRP3 inflammasome and IL-1β is important in the pathogenesis of gout, the interactive mechanism between MSU and cells has not been clearly elucidated. NLRP3 is a signal that induces ATP and potassium efflux through K+ channels such as P2X7 or toxins such as nigericin (Franchi et al., 2007; Pétrilli et al., 2007). Also, previous studies have reported that reactive oxygen species (ROS) production is also involved in NLRP3 inflammasome activation in response to MSU (Cassel et al., 2008; Dostert et al., 2008). Therefore, further studies on the precise signaling mechanism of the NLRP3 inflammasome and its response to MSU crystals deposition are necessary.

## Natural Extract Treatment Studies for NLRP3 Inflammasome Regulation in Gout

Recently, many studies have demonstrated the disease improvement effects of natural extractions for the treatment of gout through the regulation of NLRP3 inflammasome assembly. In this report, we present an overview of studies regarding NLRP3 inflammasome-based therapeutic development of natural extractions for gout within the recent 5 years (Table 1; Figure 1).

**TABLE 1:** Studies regarding natural extracts targeting NLRP3 inflammasome in gout.

Origin of extraction	Experimental model	Mode of action and target signal	Ref
*Mollugo pentaphylla* L. (MP)	C57BL6 mice + MSU	↓ Inflammatory paw edema and pain, TNF-α, IL-1β, NF-κB, NLRP3, ASC, Caspase-1	Lee et al. (2017)
*Cinnamomum burmanni* (Nees and T.Nees) Blume (CC)	C57BL6 mice + MSU	↓ LPS-induced sepsis, neutrophils recruitment, IL-1β, ASC oligomerization	Shin et al. (2017)
	BMDM + LPS		
*Actinidia arguta* (Siebold and Zucc.) Planch. ex Miq. (AA)	C57BL6 mice + MSU	↓ IL-1β, NLRP3, neutrophils recruitment	Heo et al. (2018)
	BMDM + LPS		
Taiwanese green propolis (TGP)	C57BL6 mice + MSU	↓ Pro IL-1β expression, NF-κB, ROS, mitochondrial damage, lysosomal rupture, (p)JNK, ASC, NLRP3, neutrophils recruitment, IL-1β, caspase-1, IL-6, MCP-1	Hsieh et al. (2019)
	J774A.1 + LPS + MSU		
	THP-1 + LPS + MSU		
	BMDM + LPS + MSU		
*Chrysanthemum indicum* L. (CI)	C57BL6 mice + MSU	↓ IL-1β, caspase-1, ASC, neutrophils recruitment	Yu et al. (2019)
	BMDM + LPS + nigericin		
*Cichorium intybus* L. (CIL)	SD rat + MSU	↓ Swelling degree, skin temperature, IL-1β, NF-κB, pIκBα, NLRP3, (p)p65	Wang et al. (2019)
	Rat macrophage		
Huzhen Tongfeng (HZTF)	SD rat + MSU	↑ Antioxidant capacity	Wu et al. (2019)
	Raw 264.7 + H2O2	↓ Swelling degree, neutrophils infiltration, IL-1β, IL-6, TNF-α, ASC	
	THP-1 + MSU		
Red ginseng (RG)	C57BL6 mice + MSU	↓ IL-1β, ASC, NLRP3, caspase-1, neutrophils recruitment, IL-6, TNF-α, TGF-β	Lund et al. (2021)
	THP-1 + LPS + MSU		
	Gout patients		

*Mollugo pentaphylla* L [Molluginaceae] (MP) is a component of an important traditional medicine in Taiwan which is used as an anticancer, antitoxic and diuretic agent. A previous study reported that MP suppressed inflammatory paw edema and pain in MSU-induced gout mice. In addition, MP showed anti-inflammatory activity by inhibiting the production of TNF-α, IL-1β, NLRP3 inflammasome and NF-κB (Lee et al., 2017).

*Cinnamomum burmanni* (Nees and T. Nees) Blume [Lauraceae] (CA) is known as a food or aroma. It has shown potential for antimicrobial activity *in vitro*, and is used to treat diarrhea, lack of energy, and shortness of breath in traditional Chinese medicine. A previous research demonstrated that CA improved the survival rate of the LPS-induced septic shock mouse model and inhibited inflammasome activation including NLRP3, NLRC4, and AIM2, leading to suppression of interleukin-1β secretion. In addition, CA attenuated ASC oligomerization and its speck formation, and also enhanced the survival rates of both LPS induced septic shock and MSU-induced gout mice models (Shin et al., 2017).

*Actinidia arguta* (Siebold and Zucc.) Planch. ex Miq [Actinidiaceae] (AA) is known to have abundant nutrients, such as vitamins, polyphenols, etc. An existing study reported that AA regulated NLRP3 ubiquitination and ASC oligomerization, leading to the inhibition of NLRP3 inflammasome-mediated IL-1β secretion in the MSU-induced gout mice model (Heo et al., 2018).

Taiwanese green propolis (TGP), a type of Taiwanese propolis, is known for its various beneficial biological functions, such as anticancer, anti-inflammatory, antifibrosis, antimicrobial, and antioxidant activities. In a prior study, TGP attenuated the peritoneal recruitment of neutrophils, and the levels of IL-1β, active caspase-1, IL-6 and MCP-1 in lavage fluids in the MSU-induced gout mice model. In addition, TGP inhibited pro IL-1β expression by reducing NF-κB activation and ROS production in LPS-activated macrophages. TGP also suppressed the activation signal by reducing mitochondrial damage, ROS production, lysosomal rupture, c-Jun N-terminal kinases 1/2 phosphorylation and ASC oligomerization. Furthermore, TGP inhibited the NLRP3 inflammasome partially via autophagy induction (Hsieh et al., 2019).

*Chrysanthemum indicum* L [Asteraceae] (CI) is a plant commonly used for food, tea and aroma. A previous study showed CI inhibited activation of NLRP3 and AIM2 inflammasomes, leading to suppression of IL-1β secretion *in vitro*. In addition, CI regulated the phosphorylation of ASC, and also suppressed secretion of proinflammatory cytokines and neutrophils recruitment in the MSU-induced mice peritonitis model (Yu et al., 2019).

*Cichorium intybus* L [Asteraceae], known as chicory, is a perennial herbaceous plant of the daisy family Asteraceae. It is commonly used as a coffee substitute and food additive. In particular, chicory is also a major crop for extracting inulin, a natural polysaccharide. In a previous research, *Cichorium intybus* L. leaves (CIL) improved the swelling degree, inflammatory activity, and histopathological lesion in MSU-injected rat ankles. In addition, CIL decreased IL-1β secretion by suppressing the NF-κB and NLRP3 signaling pathways. Similar to the *in vivo* results, IL-1β release was also inhibited by CIL and chicoric acid, a specific effective compound in *Cichorium intybus* L, through the NF-κB and NLRP3 signaling pathways (Wang et al., 2019).

Huzhen Tongfeng Formula (HZTF) is a compound extract from four Chinese medical herbs: Polygoni Cuspidati Rhizoma et Radix (PCRR, the root and rhizome of *Polygonum cuspidatum* Siebold. et Zucc [Polygonaceae]), Ligustri Lucidi Fructus (LLF, the fruit of *Ligustrum lucidum* W.T.Aiton [Oleaceae]), Herba Plantaginis [HP, the dried whole grass of *Plantago asiatica* L (Plantaginaceae)], and Nidus Vespae [NV, the honeycomb of Polistes olivaceus (De Geer)], Polistes Japonicus de Saussure, or Parapolybiavaria Fabricius). HZTF is used for gouty arthritis in Chinese traditional medicine. A previous study reported that HZTF suppressed paw swelling and neutrophil infiltration in intra-articular MSU-induced gout rats. HZTF also showed inhibited inflammatory cytokines (IL-1β, IL-6, and TNF-α) secretion in MSU-induced THP-1 cells and could prevent the oligomerization of ASC. Moreover, HZTF also demonstrated antioxidant capacity in cell-free and cell-base tests (Wu et al., 2019).

Red ginseng [RG, *Panax ginseng* C.A.Mey (Araliaceae)] is known to have various effects such as immunity improvement, fatigue relief, memory improvement, blood circulation improvement, antioxidation, mitigation of menopausal women’s symptoms, and anticancer. Such effects have been reported in basic experimental research as well as clinical study. In a previous study, RG lowered the number of WBCs in the lavage fluid leading to suppress acute gout inflammation in the air-pouch mice model. Also, RG inhibited MSU-induced IL-1β production in THP-1 cells. The assembly of NLRP3 inflammasomes was inhibited via reduced ASC expression and oligomerization. Furthermore, RG treatment for 3 months in patients showed reduced NLRP3 expression compared to baseline (Lund et al., 2021).

## Conclusion

Gout is the most common form of inflammatory arthritis worldwide. The risk of gout increases with age and is therefore more common in an aging population. Allopurinol or Febuxostat has been used as treatment for gout which has been traditionally administered to lower uric acid levels. However, such traditional therapy is difficult to conduct in patients with certain underlying diseases or for long-term periods due to the risk of side effects. Previous animal model studies using anakinra (IL-1R antagonist) suggested the possibility of targeting IL-1β to modulate MSU-induced inflammation (Martin et al., 2009; Torres et al., 2009), and early clinical studies also demonstrated efficacy in the treatment of acute and chronic gout patients (Mcgonagle et al., 2007; So et al., 2007; McGonagle et al., 2008). With the increased understanding of the pathophysiology of gout, new methods of gout treatment in relation to the inflammatory process have been introduced. A previous study also showed that caffeic acid, *β*-carotene and sulforaphane modulate inflammation by acting as selective and direct inhibitors of the NLRP3 inflammasome in MSU-induced gout mice (Lee et al., 2016; Yang et al., 2018; Yang et al., 2020). This could be evidence of a new pharmacological strategy of anti-NLRP3 inflammasome activators in the prevention and treatment of gout onset.

Various studies have been conducted on natural products that inhibit gout by targeting the NLRP3 inflammasome. In this report, studies of on natural extracts using animal models over the past 5 years were summarized and presented. Such an overview suggests that the use of natural products acting at various stages of NLRP3 signaling may be a suitable pharmacological approach for the management of acute and chronic gout.

However, despite great advances in our understanding of the inflammatory processes associated with gout, various issues still remain to be addressed. While the exact mechanism by which MSU crystals are recognized by immune cells remains elusive, the role of other cytokines that may be activated by the NLRP3 inflammasome, such as IL-18, must also be considered. In addition, MSU can induce inflammatory responses through pathways other than the NLRP3 inflammasome. Thus, it is important to identify these pathways and understand their interactions with the NLRP3 inflammasome and IL-1β production during the development and resolution phases of gout. Understanding such mechanisms offers us new opportunities to disrupt the inflammatory pathway and anticipate the development of more effective treatments for gout. Therefore, further research on natural products and their regulation of NLRP3 inflammasome assembly in a wide range of gout is needed to elucidate various pharmacological mechanisms and develop effective gout therapeutics.
